# Medication and Nutrition by Absorption

**Published:** 1880-01

**Authors:** E. H. Gibbs


### MEDICATION AND NUTRITION BY ABSORPTION.

BY E. H. GIBBS, A. M., M. D.

(*Editor of Hall's Journal of Health.*)

I have no doubt that nourishment might be introduced into the system through the skin, in quantities sufficient to sustain life for a long time. There is no better or more effectual method of introducing alterative medicines, such as mercurial ointment, as all physicians are aware, than by rubbing them into the skin. This being the case, how much more effectual is the plan of introducing medicines and appropriate foods into the rectum, since mucous membranes absorb far more readily and rapidly than the skin, and, besides, the rectum has considerable digestive power.

I have to acknowledge my indebtedness to Dr. Wm. Bodenhamer, author of that admirable little book entitled "AN ESSAY ON RECTAL MEDICATION," for many important facts relating to this subject. In SECTION 1, under the head of

GENERAL REMARKS, he says:

"In the employment of an officinal therapeutic remedy, it is not only important and necessary to know in what case, in what dose, in what form, but also *by what channel* it should be administered, for a great point or desideratum in the use of all medicines, but more especially those that operate through the medium of absorption, is their rapid transmission, pure reception into the system, and their speedy and efficient effect, both local and remote. In many instances, the principal object may be to allay excitement, to give ease, and to procure rest or sleep as soon as possible. The stomach may be too irritable to tolerate medicine or anything else, or in such a condition as to be incapable of absorbing it; or to do this very slowly and imperfectly, as is frequently the case. Some medicines when addressed to the stomach, tend to impair digestion at a time when it is highly important to improve it, or, at least, not to interfere with it; whereas the same medicine, if it were administered *per rectum*, would not, perhaps, in the least, interfere with digestion. Some medicines, too, are very liable to become decomposed or contaminated in the stomach. Patients frequently refuse to swallow medicine, or are unable to do so. These several circumstances, with many others that might be named, have pointed to the necessity, in such instances, of a somewhat more certain, speedy, and efficient method than the ordinary one of stomachic medication."

HYPODERMIC MEDICATION

is referred to on page 6 thus: "This now very common and often misused method is of great and undoubted value in numerous cases, yet I am decidedly of opinion that rectal medication is of greater importance; indeed, it is greatly preferable in many instances, being much less dangerous, much more convenient and simple; and the remedies, too, act through this medium with almost equal celerity, as I have often verified. But very few remedies can be used hypodermically in comparison to those which can be used by the rectum; and, furthermore, the certainty of rapidly introducing into the system, *per rectum*, the most active narcotics and other remedies, without having to resort to the operation of hypodermic injection, will prove valuable in those cases in which an operation, however inconsiderable, is always more or less dreaded, and also in cases in which it may be found highly necessary to keep the patient for a considerable time under the influence of the remedy, by frequently repeating it, as in persistent neuralgia, puerperal mania, mania-a-potu, hydrophobia, tetanus, etc. I have seen cases in which the operation of hypodermic injection had been so often repeated that all the eligible places of the body for the puncture were covered with cicatrices so as to leave no place for new punctures except through the old ones. Another serious objection to hypodermic injections is the great liability to the formation of troublesome abscesses in the tegumentary walls."

The absorption of medicines, when introduced into the rectum, is far more rapid than when taken into the stomach, and their effect is more regular and more equable; since medicines taken into the stomach become mingled with its contents, and before reaching the general circulation, must pass through the portal system.

### RECTAL ABSORPTION.

"The mucous membrane of the re[illegible]m, like that of other portions of the alimentary canal, possesses all the requisites, more or less, for the exercise of absorption. Any one can assure himself at once, not only of the power of the rectum to absorb, but also of the rapidity of that act, by injecting four or eight ounces of warm linseed tea, or barley-water, into the rectum, and retaining it for a few hours; and at the end of that time, should an evacuation of the bowels take place, not a drop, perhaps, of the injected fluid would be found in the fæcal dejection. Liebig states that a solution of common salt, in the proportion of one part of the salt to eighty parts of water, disappeared so completely in the rectum, that an evacuation one hour afterward was found to contain no more than the usual portion of salt.

The absorbents of the rectum are much more numerous than those of any other portion of the large intestine, and it has a larger number of blood vessels. It is the only portion of the alimentary canal which is supplied with nerves directly from the great source of motion and sensation, the spinal marrow. This explains why many medicinal substances act more energetically when applied to the rectum than when received into the stomach; and likewise it tends to explain the principles upon which such remedies act in relieving painful affections of the rectum and adjacent organs. On page 29, Bodenhamer remarks that numerous examples could here be given to prove that the rectum and the colon are capable, under certain conditions, of digesting, absorbing, or imbibing sufficient nourishment to maintain life, if properly-prepared nutriment is addressed to them. Nutritious substances are taken up by the absorbents, and by them conveyed to the *receptaculum chyli*, and thus soon find their way into the economy, and produce their assimilative and nutritive effect. Haller demonstrated the fact that lymphatic vessels existed along the whole tract of the colon and rectum; and in numerous instances chyle was found in these vessels instead of lymph, proving that digestion can and does take place in the alimentary canal. I have the greatest confidence in the absorptive and digestive powers of the rectum to sustain life indefinitely.

### "REST TO THE STOMACH,"

says Mr. Theodore Williams (in London *Lancet*, Oct. 24, 1874), "is of the highest importance in some of the diseases of this organ. In such cases, we possess in the rectum an effective second stomach, which, if it does not afford us the pleasure of digestion, spares us many of its pains."

Baron Dupuytren said : "The rectum *absorbs*, but does not *digest*. The medicinal agent," he says, "owing to the absence of digestion, passes more directly, more purely, and more surely to its destination, than the same medicine does when taken into the stomach."

M. Orfila asserts that those medical agents which operate through the medium of absorption, such as opium, tobacco, etc., are much more active by the rectum than by the stomach, and assigns as a reason the greater venous absorption of the rectum, and its less digestive power.—(*Medicine Légale*, Paris, 1821.)

It has been established beyond all doubt by Mr. W. S. Savory that strychnine is absorbed much quicker by the mucous membrane of the rectum than by the stomach ; that a small dose will act with greater energy in the rectum than a much larger dose in the stomach.—(*London Lancet*, 1866.) Bodenhamer says (page 33): "Through the medium of the rectum the most decided impressions may be transmitted by medicines to the various important viscera contiguous to it, and contained in the pelvis, the uterus, the vagina, the bladder, the prostate gland, the urethra, the seminal vesicles, &c.

Dr. Brown-Sequard (London *Lancet*, 1866) says that he has found that belladonna and opium, employed against neuralgic and other uterine pains, act with greater rapidity and much more benefit when inserted in the rectum than in the vagina, showing that absorption is more rapid by the mucous membrane of the rectum than by the vagina.

Prof. F. Barker, in cases of menorrhagia associated with the climacteric period, recommends rectal suppositories containing the aqueous extract of ergot.

Dr. Upshur reports a complete cure of small villous tumor at the margin of the internal sphincter muscle by the use, twice daily, of suppositories containing 20 grains each of extract of ergot.

### CHLORAL HYDRATE.

Dr. J. W. Hickman says (*Medical Record*, Jan. 10, 1880): "No practitioner who keeps pace with the progress of the day would think of exhibiting chloral, where either the respiration or circulation was seriously compromised. It would be absolutely criminal to give it in disease of the lungs, where the right heart is being continually overtaxed ; or in the sleeplessness of valvular disease of the heart. A fatty heart would not brook even a medium dose of chloral." J. Crichton Browne says : "Chloral exerts an effect proportional to cerebral development ; that it must be administered most cautiously to the most intellectual. Again, the matter of bodily temperature should have much to do in determining the proper dose, for serious symptoms—and even death—have occurred from neglect of this consideration. Even the temperature of the room should be taken into account ; for the tendency of chloral is to retard the heart's action."

### USES OF CHLORAL HYDRATE.

The late Prof. Biddle says : "Chloral is a most reliable hypnotic, second only in this particular to opium." It does not induce the swimmy, broken sleep of opium, but quiet, dreamless, and refreshing slumber, strikingly resembling that of health. The patient may be awakened with a confidence that he will at once relapse into forgetfulness. Nor is it, like opium, followed by disagreeable sequences, either cerebral or gastric. On the contrary, so far as the stomach is concerned, digestion is often stimulated by its use. It will not, however, exert the slightest soporific influence in painful conditions of the system; it possesses no anodyne properties whatever.

When the object is to lull pain and induce sleep, about one-fourth of a grain of morphia sulph. with ten grains of chloral will be found a most valuable combination.

Where the patient is *too weak to sleep*, anodynes will be found to aggravate rather than secure rest; while in chloral, perhaps, with the bromides given with a little light wine or champagne, we have a most efficient means at our command. In the sleeplessness due to excessive mental work, chloral exercises a peculiarly happy influence.

Macnamara lays great stress on its value in tetanus. Out of twenty cases in India, all traumatic, seventeen recovered by the use of chloral.

In sea-sickness, no agent has given such satisfactory results as chloral. The usual dose is about twenty grains every three or four hours, the patient observing the recumbent posture, and taking nourishment in some shape. Bartholow says: "There is no remedy now known so efficient in cholera or cholera morbus. In these cases, the remedy is enhanced in value if a little morphia is combined." Dr. Curci states (in *The Practitioner*, for October, 1879), that chloral is a most valuable remedy in dysentery.

Dr. J. H. Scorff reports four cases where he was successful in removing the vomiting of pregnancy, by introducing 20 grains of chloral into the rectum, night and morning. He only found it necessary to use the remedy three or four times.

Chloral is the remedy *par excellence* in the ravings of delirium tremens, and in cases of delirium and insomnia combined.

Infantile convulsions may frequently be suspended, by the discreet use of chloral.

Attacks of laryngismus stridulus are speedily broken up by timely doses of chloral.

Dr. Hickman reports a case of typho-malarial fever, which chloral was found to control in a happy manner. The chloral was introduced per rectum. It will generally be found safer, more effective, and easier to introduce chloral into the system per rectum. The best preparation of chloral for this purpose is the solid, or that in the form of crystals. The No. 3 Hollow Suppository will contain 20, more or less, grains of these crystals. They may be put in dry, or dissolved in water, oil, or glycerine.

Suppositories are in all respects the best vehicles for the introduction of medicines or nutriment into the rectum; but two almost insuperable obstacles have, until recently, prevented their employment to the extent they deserve.

1st. The difficulty most druggists have experienced in combining the medicine with the butter of cacao.

2d. The difficulty of making suppositories smooth, shapely. and firm, so that they may be easily inserted without breaking or crumbling.

The recent invention of HOLLOW SUPPOSITORIES of various sizes and calibres, made from butter of cacao, into which any kind of medicine or nutriment can be put, and then sealed up by means of a stopper of butter of cacao, when they will be ready for use; is a great boon to Physicians and Druggists, and must cause the general adoption by physicians of this most desirable method of medication and nutrition.

